# Preservation of carbon dioxide clathrate hydrate in the presence of trehalose under freezer conditions

**DOI:** 10.1038/srep19354

**Published:** 2016-01-19

**Authors:** Hironori D. Nagashima, Satoshi Takeya, Tsutomu Uchida, Ryo Ohmura

**Affiliations:** 1 Department of Mechanical Engineering, Keio University, 3-14-1 Hiyoshi, Kohoku-ku, Yokohama, Kanagawa 223-8522, Japan; 2 National Institute of Advanced Industrial Science and Technology (AIST), 1-1-1 Higashi, Tsukuba, Ibaraki 305-8565, Japan; 3 Faculty of Engineering, Hokkaido University, N13 W8 Kita-ku, Sapporo, Hokkaido 060-8628, Japan

## Abstract

To investigate the preservation of CO_2_ clathrate hydrate in the presence of sugar for the novel frozen dessert, mass fractions of CO_2_ clathrate hydrate in CO_2_ clathrate hydrate samples coexisting with trehalose were intermittently measured. The samples were prepared from trehalose aqueous solution with trehalose mass fractions of 0.05 and 0.10 at 3.0 MPa and 276.2 K. The samples having particle sizes of 1.0 mm and 5.6–8.0 mm were stored at 243.2 K and 253.2 K for three weeks under atmospheric pressure. The mass fractions of CO_2_ clathrate hydrate in the samples were 0.87–0.97 before the preservation, and CO_2_ clathrate hydrate still remained 0.56–0.76 in the mass fractions for 5.6–8.0 mm samples and 0.37–0.55 for 1.0 mm samples after the preservation. The preservation in the trehalose system was better than in the sucrose system and comparable to that in the pure CO_2_ clathrate hydrate system. This comparison indicates that trehalose is a more suitable sugar for the novel frozen carbonated dessert using CO_2_ clathrate hydrate than sucrose in terms of CO_2_ concentration in the dessert. It is inferred that existence of aqueous solution in the samples is a significant factor of the preservation of CO_2_ clathrate hydrate in the presence of sugar.

Carbonated drinks, *e.g.* soda, beer, champagne *etc.*, are popular all over the world because they have a feeling of refreshment. Solid carbonated foods, like a candy or jelly, are also available in markets. However, the number of solid carbonated foods products is less than that of carbonated drinks and the solid foods are not as popular as carbonated drinks because CO_2_ solubility in ice or other solid is extremely less than the solubility in liquid water. One of the solutions of this problem is clathrate hydrate.

Clathrate hydrate, also called gas hydrate, is a crystalline solid composed of water (host molecule) and other molecules (guest molecule), *e.g.* methane, ethane, carbon dioxide *etc*. Guest molecules are trapped in the molecular-level cages that are composed of hydrogen-bonded water molecules. Clathrate hydrate can store large amount of gas molecules. For example, CO_2_ concentration in clathrate hydrate is 298 kg/m^3^^1^, which is 20–50 times higher than that in carbonated water, 6–15 kg/m^3^^2^. Then, clathrate hydrate has a great potential for the solid carbonated food.

In general, high pressure and low temperature condition is necessary to thermodynamically stabilize clathrate hydrate. For example, phase equilibrium pressure of CO_2_ clathrate hydrate is 1.318 MPa at 273.8 K^3^. Preservation of CO_2_ clathrate hydrate under thermodynamically stable condition is often practically difficult because such high pressure is needed. This difficulty can be overcome using a unique phenomenon observed for clathrate hydrate that is called “self-preservation” or “anomalous preservation”^4^,^5^,^6^,^7^. Some kinds of clathrate hydrate anomalously slowly decompose below the water freezing temperature despite under their thermodynamically unstable conditions. It is suggested that the anomalous preservation phenomenon is appeared by an ice layer covering clathrate hydrate particles which is formed because of clathrate hydrate dissociation^5^,^6^. Ice plays an important role for the anomalous preservation of clathrate hydrate. Numerous observations of decomposition of CO_2_ clathrate hydrate are reported^7^,^8^. Takeya and Ripmeester^7^ reported that decomposition rate of CO_2_ clathrate hydrate decreased at the temperatures between from 220 K to 260 K under atmospheric pressure. CO_2_ clathrate hydrate showed the anomalous preservation. Sun *et al*.^8^ reported the preservation of CO_2_ clathrate hydrate samples at 253 K and 258 K under atmospheric pressure for three weeks. After the three-week preservation, CO_2_ concentration in the samples exceeded that in carbonated water. CO_2_ clathrate hydrate can be preserved at thermodynamically unstable temperature under atmospheric pressure below the water freezing temperature.

Using the anomalous preservation, CO_2_ clathrate hydrate is focused as a novel carbonated food^8^,^9^,^10^,^11^,^12^,^13^. Peters *et al*. reported a rapid production process of the novel carbonated dessert using CO_2_ clathrate hydrate^9^, a safety test for a model of the package of the dessert^10^ and a sensory test for the dessert^11^. Makiya *et al*.^12^ reported the formation pressure and temperature of clathrate hydrate which is formed from CO_2_ + ethanol at temperature from 254 K to 268 K. Sato *et al*.^13^ reported the preservation of CO_2_ clathrate hydrate samples coexisting with sucrose at 253 K and 258 K under atmospheric pressure for three weeks. After the three-week preservation, CO_2_ concentration in the samples coexisting with sucrose exceeded that in carbonated water. However, the concentration is lower than that in pure CO_2_ clathrate hydrate samples because of presence of concentrated sucrose aqueous solution in the samples^13^. This report indicates that higher CO_2_ concentration in novel carbonated desserts using CO_2_ clathrate hydrate can be achieved.

One of the candidates for the sugar is trehalose. Trehalose is a disaccharide of glucose and eutectic temperature in water-trehalose system is 270.7 K^14^. Then, ice and trehalose dihydrate phases are stable in water-trehalose system at 253 K and 258 K under atmospheric pressure, *i.e.* domestic freezer conditions. Therefore, CO_2_ concentration in CO_2_ clathrate hydrate samples coexisting with trehalose after three week preservation may exceed the concentration in the samples coexisting with sucrose and that in carbonated water. Trehalose has been used in a variety of research applications and commercial products^15^. Especially in food industry, trehalose has unique characteristics; keeping vegetables and fruits fresh, protecting vitamins against heating, protecting food products against low-temperature and freezing stress, *etc.*^15^. Trehalose may have strong advantages as a sugar for the dessert using CO_2_ clathrate hydrate.

In the present study, to investigate the preservation of CO_2_ clathrate hydrate in the presence of trehalose, mass fractions of CO_2_ clathrate hydrate in CO_2_ clathrate hydrate samples coexisting with trehalose were intermittently measured by mass measurement of CO_2_ in the samples for three weeks. The samples were prepared from trehalose aqueous solution with trehalose mass fractions of 0.05 and 0.10 at 3.0 MPa and 276.2 K. Particle sizes of the samples were 1.0 mm and 5.6–8.0 mm. These values correspond to the sizes of the ice particles in frozen dessert such as sorbet, smoothie, and frozen drink. The prepared samples were stored at 243.2 K and 253.2 K for three weeks under atmospheric pressure. We also performed powder X-ray diffraction (PXRD) measurements to identify the structures of crystalline compounds in the samples after the preservation and to calculate the mass fractions of CO_2_ clathrate hydrate and ice in the samples after the preservation. The mass fractions of CO_2_ clathrate hydrate and ice obtained from PXRD measurements were independently determined from the mass measurements.

## Results and Discussion

The CO_2_ clathrate hydrate samples prepared from trehalose aqueous solution with 0.05 and 0.10 trehalose mass fractions at 276.2 K and 3.0 MPa were stored for three weeks at 243.2 K and 253.2 K under atmospheric pressure. Particle sizes of the samples were 1.0 mm and 5.6–8.0 mm. The mass fractions of CO_2_ clathrate hydrate in the samples were 0.87–0.94 and 0.93–0.97 for the samples with 0.10 and 0.05 trehalose mass fractions before the preservation experiments, respectively. Therefore, the amount of CO_2_ clathrate hydrate and trehalose in the samples was greater than 0.97 in mass fraction. This indicates that most of the water was converted to CO_2_ clathrate hydrate.

**Figure 1:**
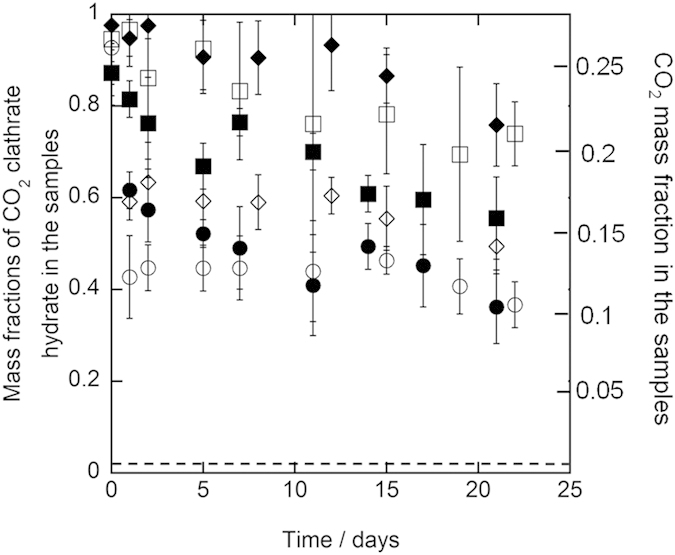
Results of the preservation of CO_2_ clathrate hydrate samples coexisting with trehalose at 243.2 K under atmosphere pressure. The plot shapes show the diameter of the CO_2_ clathrate hydrate samples and trehalose mass fraction as below, ○: 1.0 mm and 0.1 mass fraction (run 1), ◻: 1.0 mm and 0.1 mass fraction (run 2), ◻: 5.6–8.0 mm and 0.1 mass fraction (run 1), ◼: 5.6–8.0 mm and 0.1 mass fraction (run 2), ⋄: 1.0 mm and 0.05 mass fraction, ♦: 5.6–8.0 mm and 0.05 mass fraction. The dashed line indicates the mass fraction of CO_2_ clathrate hydrate, which is equivalent to carbonated water^2^. The error bar is expressed the combined uncertainty of mass fraction due to the uncertainty of the measurements and standard deviation of measured mass fractions (*n* = 3).

**Figure 2:**
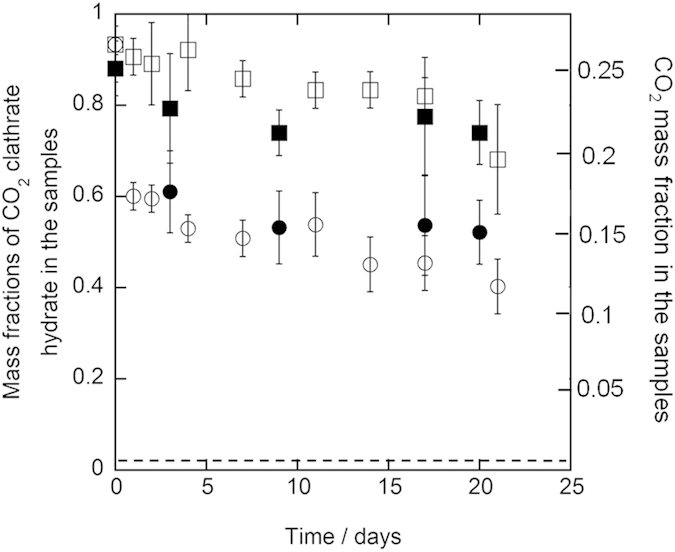
Results of the preservation of CO_2_ clathrate hydrate samples coexisting with trehalose at 253.2 K under atmosphere pressure. The plot shapes show the diameter of the CO_2_ clathrate hydrate samples and trehalose mass fraction as below, ○: 1.0 mm and 0.05 mass fraction, ◻: 5.6–8.0 mm and 0.05 mass fraction, ●: 1.0 mm and 0.1 mass fraction, ◼: 5.6–8.0 mm and 0.1 mass fraction. The dashed line indicates the mass fraction of CO_2_ clathrate hydrate, which is equivalent to carbonated water^2^. The error bar is expressed the combined uncertainty of mass fraction due to the uncertainty of the measurements and standard deviation of measured mass fractions (*n* = 3).

Results of the three-week preservation experiments for all samples at 243.2 K and 253.2 K are shown in Figs 1 and 2, respectively. In the first two days of the preservation, the mass fractions decreased from 0.87–0.97 to 0.40–0.61 for 1.0 mm diameter samples and to 0.79–0.95 for 5.6–8.0 mm diameter samples at both 243.2 K and 253.2 K. Afterwards, the mass fractions still remained around 0.4–0.6 for 1.0 mm diameter samples and around 0.6–0.9 for 5.6–8.0 mm diameter samples during the preservation. At the end of the preservation, the mass fractions still remained 0.37–0.55 for 1.0 mm diameter samples and 0.56–0.76 for 5.6–8.0 mm diameter samples. The mass fractions of CO_2_ clathrate hydrate in the samples after the three-week preservation are specified in Table 1. All of the mass fractions after the preservation significantly exceeded 0.02 that is equivalent to carbonated water^2^. This indicates that CO_2_ clathrate hydrate in the samples was anomalously preserved after the preservation although the samples were stored under thermodynamically unstable conditions of CO_2_ clathrate hydrate. The mass fractions in 5.6–8.0 mm diameter samples were higher than those in 1.0 mm diameter samples after the preservation at both 243.2 K and 253.2 K. This trend is consistent with the results of the pure CO_2_ clathrate hydrate preservation^8^. In contrast, the mass fractions after the preservation have not changed depending on the trehalose mass fraction and the preservation temperature in the present preservation experiments.

**Figure 3:**
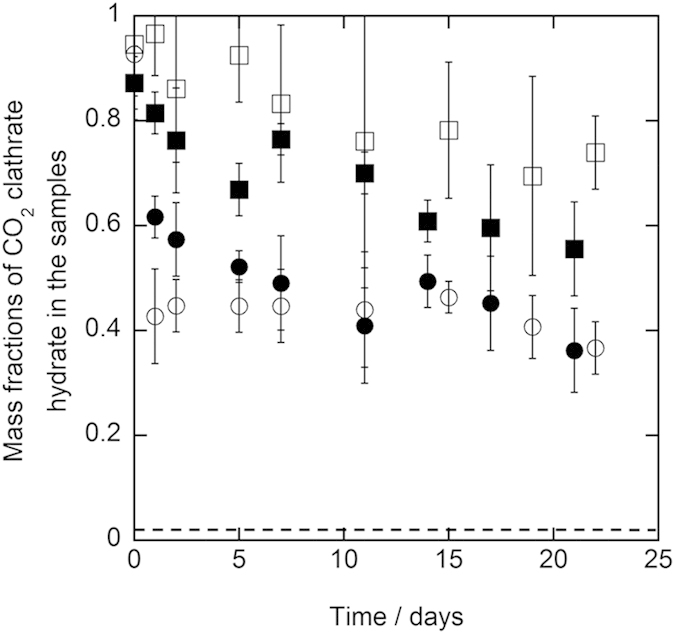
Results of the duplicate preservation experiment of CO_2_ clathrate hydrate samples coexisting with trehalose at 243.2 K under atmosphere pressure. The plot shapes show the diameter of the CO_2_ clathrate hydrate samples and trehalose mass fraction as below, ○: 1.0 mm and 0.1 mass fraction (run 1), ●: 1.0 mm and 0.1 mass fraction (run 2), ◻: 5.6–8.0 mm and 0.1 mass fraction (run 1), ◼: 5.6–8.0 mm and 0.1 mass fraction (run 2). The dashed line indicates the mass fraction of CO_2_ clathrate hydrate, which is equivalent to carbonated water^2^. The error bar is expressed the combined uncertainty of mass fraction due to the uncertainty of the measurements and standard deviation of measured mass fractions (*n* = 3).

Duplicate preservation experiments were performed to confirm the reproducibility of the preservation experiments for the samples with 0.10 trehalose mass fraction at 243.2 K. The results of the two independent runs were shown in Fig. 3 as circle and square plots for 1.0 mm and 5.6–8.0 mm diameter samples, respectively. The results of both runs were consistent for each particle size of the samples within the combined uncertainty of the mass fraction of CO_2_ clathrate hydrate. This consistency supports the reproducibility of the preservation of CO_2_ clathrate hydrate in the presence of trehalose. Both difference of the mass fractions between two runs and deviation of each mass fraction for 1.0 mm diameter samples tended to be smaller than those for 5.6–8.0 mm diameter samples. This trend indicates that distribution of CO_2_ clathrate hydrate among the sample particles for 1.0 mm diameter samples were more homogeneous than that for 5.6–8.0 mm diameter samples.

**Figure 4:**
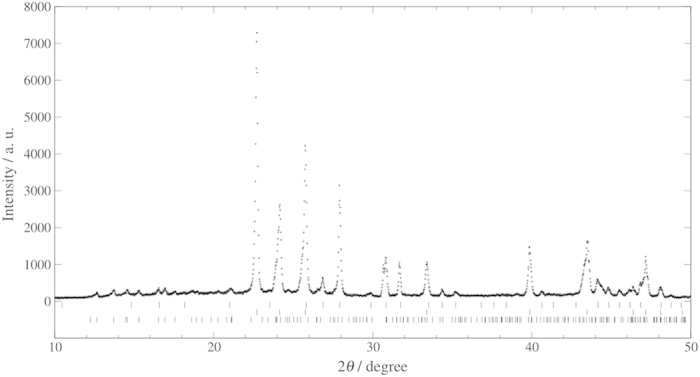
Powder X-ray diffraction pattern obtained after the three-week preservation at 243.2 K from CO_2_ clathrate hydrate samples coexisting with trehalose which were prepared from trehalose aqueous solution with 0.10 mass fraction of trehalose and preserved for three weeks at 243.2 K under atmosphere. The tick patterns correspond to structure I clathrate hydrate, hexagonal ice and trehalose dihydrate from the top, respectively.

**Figure 5:**
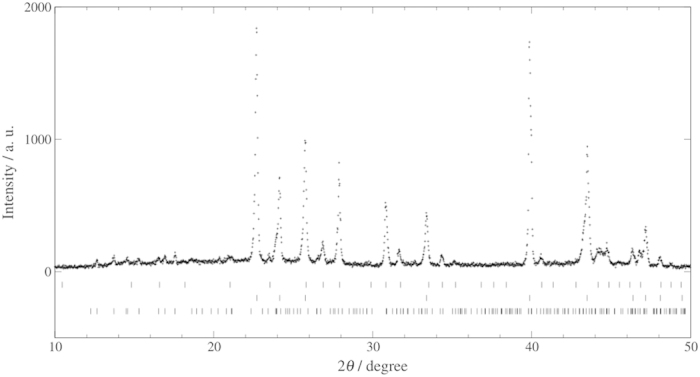
Powder X-ray diffraction pattern obtained after the three-week preservation at 253.2 K from CO_2_ clathrate hydrate samples coexisting with trehalose which were prepared from trehalose aqueous solution with 0.10 mass fraction of trehalose and preserved for three weeks at 253.2 K under atmosphere. The tick patterns correspond to structure I clathrate hydrate, hexagonal ice and trehalose dihydrate from the top, respectively.

We performed PXRD measurements to identify the crystallographic structures of crystalline compounds in the samples after the three-week preservation. Figures 4 and 5 show PXRD peak patterns obtained with the 1.0 mm diameter samples prepared from 0.10 mass fraction trehalose solution and both stored and measured at 243.2 K and 253.2 K, respectively. In both of the samples, hexagonal ice, structure I clathrate hydrate and trehalose dihydrate were observed. The existence of hexagonal ice and trehalose dihydrate in the samples is reasonable because the eutectic point temperature and composition of water-trehalose system were reported to be 270.7 K and 0.298 mass fraction of trehalose, respectively^14^. Preservation of CO_2_ clathrate hydrate in the samples coexisting with trehalose was confirmed by PXRD measurements. We also performed PXRD measurements to calculate the mass fractions of CO_2_ clathrate hydrate and ice in the samples with 0.10 trehalose mass fraction after the preservation at 100 K by the Rietveld method using RIETAN-FP program^16^. The mass fractions of CO_2_ clathrate hydrate and ice were independently determined from the mass measurements. The mass fractions of CO_2_ clathrate hydrate and ice in the samples after the preservation were calculated to be 0.28 and 0.62 for the 1.0 mm diameter samples stored at 243.2 K, and 0.37 and 0.53 for the 1.0 mm diameter samples stored at 253.2 K, respectively. The mass fractions of CO_2_ clathrate hydrate in the samples obtained from PXRD measurements were approximately 0.1 lower than those obtained from the mass measurements. This difference may be due to the decomposition of CO_2_ clathrate hydrate during powdering process of PXRD measurements. The mass fractions of hexagonal ice in the samples after the preservation were approximately equal to decreases in the mass fractions of CO_2_ clathrate hydrate in the samples obtained from the mass measurements. It indicates that most of the hexagonal ice was produced by decomposition of CO_2_ clathrate hydrate during the preservation.

**Figure 6:**
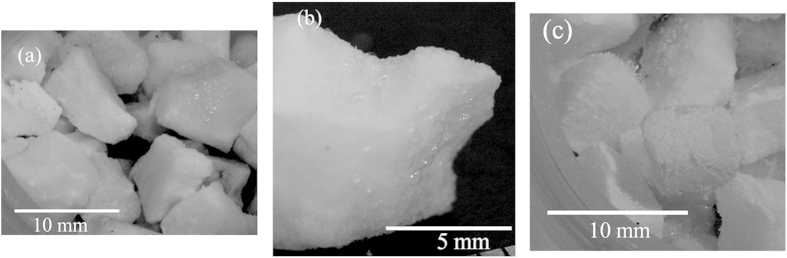
Visual images of the surfaces of the 5.6–8.0 mm diameter CO_2_ clathrate hydrate samples coexisting with 0.10 mass fraction of trehalose. (**a**) Before the three-week preservation at 253.2 K, (**b**) Focused on a particle shown in Fig. 8a, (**c**) After the three-week preservation at 253.2 K. These pictures were taken at 253.2 K.

We visually observed the surfaces of the samples before and after the preservation experiments. Figure 6 shows the surfaces of 5.6–8.0 mm diameter samples prepared from 0.10 trehalose mass fraction aqueous solution. Both the observations and the preservation were performed at 253.2 K. Before the preservation, the surfaces were smooth as shown in Fig. 6a and some bubbles were observed at the surfaces as shown in Fig. 6b. Because the bubbles were observed at the surfaces, liquid and gas existed at the surfaces. It is reported that pure CO_2_ clathrate hydrate decomposed to metastable super-cooled water below the water freezing temperature^17^. Although CO_2_ solubility in trehalose aqueous solution was not found in the literature, it is reported that CO_2_ solubility both in sucrose aqueous solution^18^ and in glucose aqueous solution^19^ were lower than that in pure water and decreased with an increase in concentration of sucrose and glucose in solution. CO_2_ solubility in the sucrose solution with sucrose mass fraction of 0.12^18^ and in the glucose solution with glucose mass fraction of 0.12^19^ are approximate 10% and 20% lower than that in pure water, respectively. Thus, the effect of CO_2_ in trehalose aqueous solution on the depression of water freezing temperature may be approximately the same as or lower than that in pure water. The depression of water freezing temperature by CO_2_ in trehalose aqueous solution is estimated to be up to 0.2 K under 0.1 MPa of CO_2_^2^. Thus, trehalose aqueous solution is thermodynamically unstable at 253.2 K^14^. The liquid observed at the surfaces is regarded as super-cooled water or super-cooled trehalose aqueous solution. After the preservation experiments, the surfaces changed to powder-like and the edges of the samples became round as shown in Fig. 6c. These observed changes at the surfaces indicate that CO_2_ clathrate hydrate decomposed at the surfaces. According to the visual observation before and after the preservation, CO_2_ clathrate hydrate in the samples decomposed to super-cooled water or solution at the surfaces. Results of the PXRD measurements and the visual observation indicate that the super-cooled water or solution was transformed to hexagonal ice at the surfaces.

**Figure 7:**
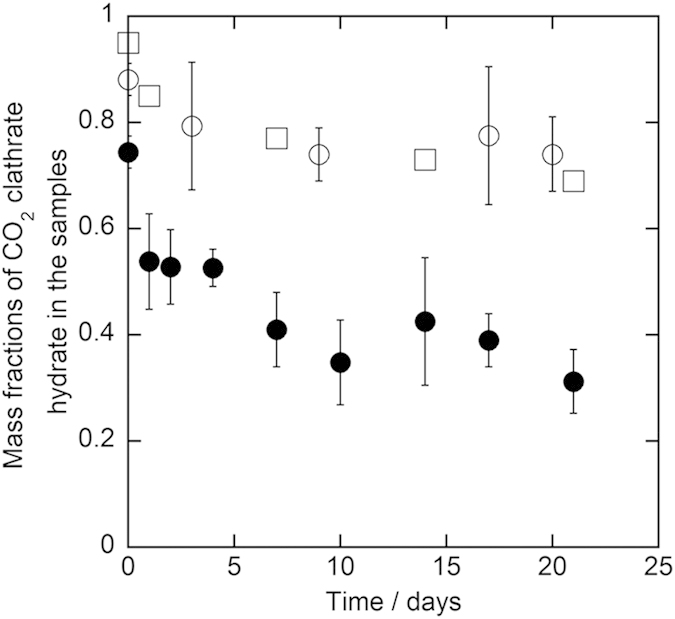
Results of the preservation of CO_2_ clathrate hydrate samples at 253.2 K under atmosphere pressure. Diameter of every sample was 5.6–8.0 mm. The plot shapes show trehalose or sucrose mass fraction as below, ○: 0.10 trehalose mass fraction, ◻: without trehalose and sucrose^8^, ●: 0.12 sucrose mass fraction^13^. The error bar is expressed the combined uncertainty of mass fraction due to the uncertainty of the measurements and standard deviation of measured mass fractions (*n* = 3).

**Figure 8:**
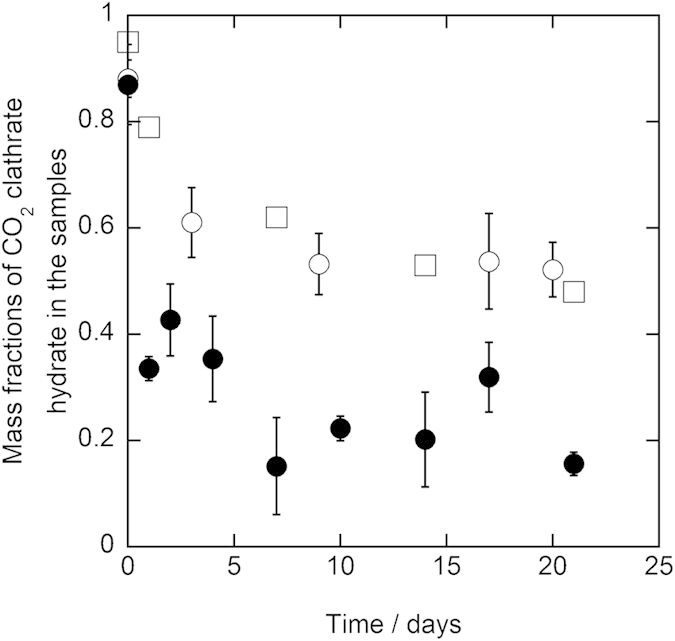
Results of the preservation of CO_2_ clathrate hydrate samples at 253.2 K under atmosphere pressure. The plot shapes show sample diameter and trehalose or sucrose mass fraction as below, ○: 1.0 mm and 0.10 trehalose mass fraction, ◻: 2.8 mm and without trehalose and sucrose^8^, **●**: 1.0 mm and 0.12 sucrose mass fraction^13^. The error bar is expressed the combined uncertainty of mass fraction due to the uncertainty of the measurements and standard deviation of measured mass fractions (*n* = 3).

Figures 7 and 8 show the comparison of the preservation in the trehalose system with the preservation in the pure CO_2_ clathrate hydrate system^8^ and in the sucrose system^13^ at 253.2 K for 5.6–8.0 mm samples and for 1.0 mm (with sucrose^13^ and trehalose) or 2.8 mm (without sugars^8^) samples, respectively. In Fig. 8, the results for two difference diameters are shown because the smallest diameter of the samples without sugars^8^ was 2.8 mm. For both 5.6–8.0 mm and 1.0 mm samples, the preservation in the trehalose system was comparable to that in the pure system^8^, whereas better than that in the sucrose system^13^. Note that the preservation for 1.0 mm samples with trehalose was comparable to that for 2.8 mm samples without sugars although the diameter of samples with trehalose was smaller than that without sugars. This comparison indicates that trehalose is a more suitable sugar for the novel carbonated dessert using CO_2_ clathrate hydrate than sucrose in terms of CO_2_ concentration in the dessert. The anomalous preservation may appear by an ice layer that is formed by dissociation of clathrate hydrate^5^,^6^ In the samples coexisting with sucrose^13^, sucrose aqueous solution may exist because the preservation temperatures were higher than the eutectic temperature of water-sucrose system. Sato *et al*.^13^ discussed that the sucrose aqueous solution in the samples influenced the ice formation and thus the preservation in the sucrose system was inferior to that in the pure system. In contrast, CO_2_ clathrate hydrate in the samples coexisting with trehalose decomposed to ice at the surfaces of the samples because the preservation temperature was lower than the eutectic temperature of trehalose-water system that is reported to be 270.7 K^14^. Better preservation with trehalose than that with sucrose^13^ may be ascribed to the absence of aqueous solution in the samples. It is inferred from the comparison that existence of aqueous solution in the samples is a significant factor of the preservation of CO_2_ clathrate hydrate in the presence of sugar.

## Methods

### Sample preparation

**Figure 9:**
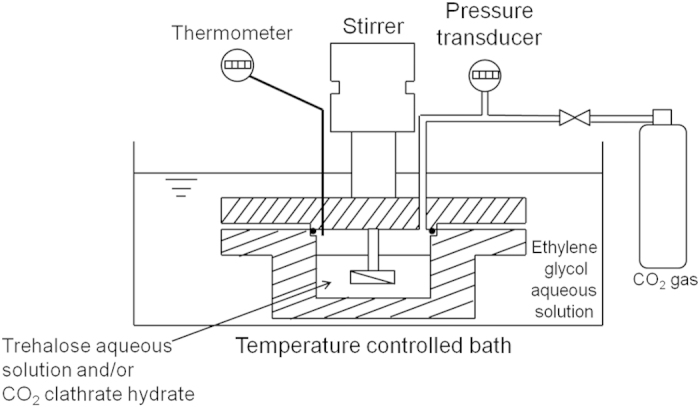
Schematic of the experimental apparatus used to prepare CO_2_ clathrate hydrate samples coexisting with trehalose.

The CO_2_ clathrate hydrate samples coexisting with trehalose were prepared in the pressure vessel as shown in Fig. 9. The inner dimensions of the vessel were diameter 80 mm, height 40 mm and volume about 200 cm^3^. The vessel was placed in a temperature-controlled bath filled with ethylene glycol aqueous solution at 276.2 K. A platinum resistance thermometer (with uncertainly of ±0.1 K) and a pressure transducer (GC31-174-L7N18, Nagano Keiki Co., Ltd., with uncertainly of ±0.05 MPa) were used to measure the temperature and pressure inside the vessel. In the vessel, an impeller was driven at 400 rpm to agitate trehalose aqueous solution, gas and CO_2_ clathrate hydrate particles. To prepare the samples, we supplied approximate 50 g of trehalose aqueous solution with trehalose mass fraction of 0.05 or 0.10 to the vessel. Trehalose dihydrate (Hayashibara Co., Ltd., 0.98 mass fraction certified purity) and laboratory-made deionized and distilled water (electrical conductivity was less than 0.5 × 10^−4^ S/m) were used. To discharge the air inside the vessel, CO_2_ gas (Toyoko Kagaku Co., Ltd., 0.999 volume fraction certified purity) was supplied to the vessel and discharged using a vacuum pump till the partial pressure of the air decreased to 0.01 kPa or lower. Then, CO_2_ gas was supplied to the vessel at 3.0 MPa and 276.2 K to form CO_2_ clathrate hydrate. This pressure is higher than the phase equilibrium pressure for CO_2_ clathrate hydrate forming system at 276.2 K, 1.717 MPa^20^. During preparing the samples, the pressure in the vessel decreased because of CO_2_ clathrate hydrate formation. Then, CO_2_ gas was recharged in the vessel at 3.0 MPa until no further pressure reduction was observed. After that, the vessel was transferred to liquid nitrogen bath and cooled to 219.2 K. During the cooling procedure, the pressure inside the vessel was reduced in a stepwise manner, approximately along the pressure-temperature line of the ice + vapor + CO_2_ clathrate hydrate equilibrium. The samples were removed from the vessel at 219 K under atmospheric pressure. The samples were immediately crushed using a chilled mortar and pestle, and sieved into particles with diameters of 1.0 mm and 5.6–8.0 mm.

### Preservation experiments

**Figure 10:**
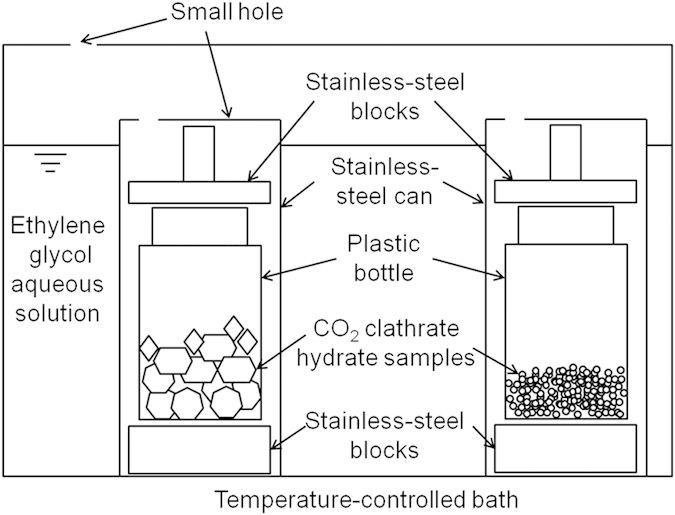
Schematic of apparatus for the three-week preservation experiment of CO_2_ clathrate hydrate samples coexisting with trehalose.

During the preservation experiments, the samples were stored in plastic bottles at 243.2 K or 253.2 K under atmospheric pressure as shown in Fig. 10. The plastic bottles were set in stainless-steel cans placed in a temperature-controlled bath and mechanically stabilized by stainless-steel blocks used as sinkers. The temperature in the bottles was controlled by ethylene glycol aqueous solution in temperature-controlled bath. The temperature inside the cans was intermittently measured using a platinum resistance thermometer to confirm that the sample temperature was kept at the prescribed value, 243.2 K or 253.2 K. During the three-week preservation, approximate 1.5 g samples were intermittently removed from the bottles. The removed samples were used to the mass measurements to determine the mass fractions of CO_2_ clathrate hydrate in the samples.

To determine the mass fractions of CO_2_ clathrate hydrate in the samples, we measured CO_2_ mass in the samples. The removed samples were divided into three portions. We placed the portions in three closed containers and measured mass of the samples in the container using an electronic balance (Sefi IUW-200D, with uncertainly of ±0.1 mg). Then, the containers were heated to 293.2 K to decompose all of CO_2_ clathrate hydrate in the samples. After the decomposition, the containers were cooled to 253.2 K, and the containers were opened once to release CO_2_ gas evolved from CO_2_ clathrate hydrate into atmosphere. After that, the containers were reheated to 293.2 K to dissociate ice in the containers and mass of the samples was measured using the electronic balance. The mass measurements and cooling were repeated until no further mass reduction was observed, that is, mass reduction was smaller than 1 mg. The difference between the first and last mass of the samples is the CO_2_ mass in the samples. The mass of CO_2_ clathrate hydrate in the samples was calculated based on the following equation using the hydration number of CO_2_ clathrate hydrate prepared at 276 K and 3.0 MPa reported by Udachin *et al*.^1^ , 6.2,
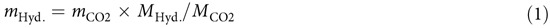
where *m*_Hyd._ is mass of CO_2_ clathrate hydrate in the samples, *m*_CO2_ is the CO_2_ mass in the samples, *M*_CO2_ is molar mass of CO_2 ,_and *M*_Hyd._ is the molar mass of CO_2_ clathrate hydrate per 1 mol of CO_2_. The mass fraction of CO_2_ clathrate hydrate in the samples was defined as *m*_Hyd._/*m*_sample_, where *m*_sample_ is mass of the samples removed from the plastic bottles. The each mass fraction of CO_2_ clathrate hydrate in the samples was calculated for the each sample portion. The average of the three measured values was taken as the mass fraction of CO_2_ clathrate hydrate in the samples. The uncertainty of the mass fraction measurements of CO_2_ clathrate hydrate in the samples was estimated to be ±0.02. The combined uncertainty of the mass fraction of CO_2_ clathrate hydrate was calculated to be the positive square root of the square-sum of the uncertainty of the mass fraction measurements and the deviation of the three measured mass fraction values.

### Powder X-ray diffraction (PXRD) measurements

We performed PXRD measurements to identify the structures of crystalline compounds in the samples and to calculate the mass fractions of CO_2_ clathrate hydrate and ice in the samples after the three-week preservation by Rietveld method using RIETAN-FP program^17^. For the identification, we used the 1 mm diameter samples and performed PXRD measurements at the preservation temperature, 243.2 K or 253.2 K. For the calculation, PXRD measurements were performed at 100 K using 1.0 mm diameter samples. The samples used in both measurements were top-loaded on a cupper (Cu) specimen holder. Both of the PXRD measurements were performed using Cu Kα radiation in *θ*/2*θ* scanning mode in the 2*θ* range 6–60°, with a step width of 0.02° (40 kV, 40 mA; Rigaku Ultima III).

## Additional Information

**How to cite this article**: Nagashima, H. D. *et al*. Preservation of carbon dioxide clathrate hydrate in the presence of trehalose under freezer conditions. *Sci. Rep.* **6**, 19354; doi: 10.1038/srep19354 (2016).

## Figures and Tables

**Table 1:** The mass fractions of CO_2_ clathrate hydrate in the CO_2_ clathrate hydrate samples coexisting with trehalose after the three-week preservation experiments with experimental conditions (trehalose mass fractions, particle sizes of the samples and preservation temperature).

**Trehalose mass fractions**	**Particle sizes/mm**	**Preservation temperature/K**	**Mass fractions of CO**_**2**_ **clathrate hydrate in the samples**
0.05	5.6–8.0	243	0.76 ± 0.09^a^
0.05	1.0	243	0.49 ± 0.06^a^
0.05	5.6–8.0	253	0.68 ± 0.11^a^
0.05	1.0	253	0.40 ± 0.06^a^
0.1	5.6–8.0	243	0.74 ± 0.07^a^ (run 1)
			0.56 ± 0.09^a^ (run 2)
0.1	1.0	243	0.37 ± 0.05^a^ (run 1)
			0.36 ± 0.06^a^ (run 2)
0.1	5.6–8.0	253	0.74 ± 0.07^a^
0.1	1.0	253	0.52 ± 0.07^a^

^a^Data are expressed as the mean ± the combined uncertainty of the mass fraction due to the uncertainty of the measurements and standard deviation of measured mass fractions (*n* = 3).
